# Comparison of Muscle Regeneration after BMSC-Conditioned Medium, Syngeneic, or Allogeneic BMSC Injection

**DOI:** 10.3390/cells11182843

**Published:** 2022-09-12

**Authors:** Barbara Świerczek-Lasek, Lukasz Tolak, Lukasz Bijoch, Marzena Stefaniuk, Patrycja Szpak, Ilona Kalaszczynska, Władysława Streminska, Maria A. Ciemerych, Karolina Archacka

**Affiliations:** 1 Department of Cytology, Institute of Developmental Biology and Biomedical Sciences, Faculty of Biology, University of Warsaw, Miecznikowa Str. 1, 02-096 Warsaw, Poland; 2 Laboratory of Neuronal Plasticity, BRAINCITY, Nencki Institute of Experimental Biology, Polish Academy of Sciences, Pasteur Str. 3, 02-093 Warsaw, Poland; 3 Laboratory of Neurobiology, BRAINCITY, Nencki Institute of Experimental Biology, Polish Academy of Sciences, Pasteur Str. 3, 02-093 Warsaw, Poland; 4 Department of Histology and Embryology, Medical University of Warsaw, Banacha Str. 1b, 02-004 Warsaw, Poland; 5 Laboratory for Cell Research and Application, Medical University of Warsaw, Banacha Str. 1b, 02-097 Warsaw, Poland

**Keywords:** skeletal muscle, regeneration, immune response, inflammation, immunomodulation, bone marrow-derived stromal cells, conditioned medium, transplantation

## Abstract

For many years optimal treatment for dysfunctional skeletal muscle characterized, for example, by impaired or limited regeneration, has been searched. Among the crucial factors enabling its development is finding the appropriate source of cells, which could participate in tissue reconstruction or serve as an immunomodulating agent (limiting immune response as well as fibrosis, that is, connective tissue formation), after transplantation to regenerating muscles. MSCs, including those derived from bone marrow, are considered for such applications in terms of their immunomodulatory properties, as their naive myogenic potential is rather limited. Injection of autologous (syngeneic) or allogeneic BMSCs has been or is currently being tested and compared in many potential clinical treatments. In the present study, we verified which approach, that is, the transplantation of either syngeneic or allogeneic BMSCs or the injection of BMSC-conditioned medium, would be the most beneficial for skeletal muscle regeneration. To properly assess the influence of the tested treatments on the inflammation, the experiments were carried out using immunocompetent mice, which allowed us to observe immune response. Combined analysis of muscle histology, immune cell infiltration, and levels of selected chemokines, cytokines, and growth factors important for muscle regeneration, showed that muscle injection with BMSC-conditioned medium is the most beneficial strategy, as it resulted in reduced inflammation and fibrosis development, together with enhanced new fiber formation, which may be related to, i.e., elevated level of IGF-1. In contrast, transplantation of allogeneic BMSCs to injured muscles resulted in a visible increase in the immune response, which hindered regeneration by promoting connective tissue formation. In comparison, syngeneic BMSC injection, although not detrimental to muscle regeneration, did not result in such significant improvement as CM injection.

## 1. Introduction

The design of efficient and safe cell therapy to improve impaired skeletal muscle function has been the focus of extensive studies for many years. In healthy individuals, skeletal muscle tissue is able to regenerate after injury, due to the pool of its stem cells—satellite cells (SCs). However, during ageing or progression of muscle wasting diseases, such as Duchenne muscular dystrophy (DMD), this regenerative potential diminishes due to the depletion of the SC pool resulting from recurring injuries and subsequent rounds of regeneration. It was initially proposed that replacing missing or nonfunctional SCs with those transplanted and functional could restore muscle structure and improve its function. Furthermore, after homing in the SC niche, transplanted cells could potentially participate in future rounds of regeneration. However, despite numerous attempts, SC transplantations were far from successful to this date [1]. This is related mainly to the fact that it is difficult to obtain a sufficient number of SCs for successful transplantation as well as to maintain their regenerative potential during in vitro propagation, as these cells are immediately activated during isolation [2,3]. Furthermore, in many attempts transplanted myoblasts were rejected a few hours after injection by the recipient’s immune system and the remaining cells migrated poorly within the recipient tissue [4]. Due to these difficulties, other sources of cells, for example stem cells, have been considered [5]. Some of them, such as pluripotent stem cells (PSCs), are undoubtedly characterized by myogenic potential, as they can convert into skeletal muscle tissue during teratoma or chimera formation [6,7,8,9]. They were also shown to be able to differentiate into functional SCs during in vitro culture when exposed to myogenic regulators, such as WNT, BMP4 inhibitor, FGF2, or when factors crucial for embryonic myogenesis were overexpressed in these cells [10,11]. Some studies also showed that these stem cell derivatives were able to participate in muscle regeneration in vivo and repopulate the SC niche [12,13]. However, although presenting proof of concept, these in vivo studies were performed using immunodeficient mice, such as *Rag2^−/−^γC^−/−^* or *Rag1^−/−^* [12,13]. Therefore, they did not reflect some difficulties associated with stem cell therapies, such as possible immune rejection. This is an important issue, as other studies on PSC derivatives suggested that the immune system can reject these cells, even during autologous transplantation [14]. Therefore, the presence of such cells in damaged muscle may lead to prolonged inflammation, which could eventually lead to progressive muscle weakness, muscle atrophy, and damage to neighboring structures, such as the vasculature [15]. Graft rejection could be circumvented with the application of immunosuppression, but known immunosuppressive agents can also affect skeletal muscles by blocking myoblast fusion or causing muscle atrophy [16] (Świerczek-Lasek et al., in preparation).

As an alternative to traditional immunosuppression, mesenchymal stromal cells (MSCs), such as bone marrow stromal cells (BMSCs), are considered. MSCs can block the activation and proliferation of B and T cells, dendritic cells, and NK cytotoxic cells [17]. These properties of MSCs have already been used as a treatment for several diseases. For example, allogeneic BMSCs were used as a graft versus host disease (GvHD) treatment in Canada (Prochymal). In addition, various MSCs derived from different sources are currently being tested as a potential treatment for diseases, such as GvHD, myocardial infarction, stroke, Crohn’s disease, multiple sclerosis, amyotrophic lateral sclerosis, diabetes, lupus, arthritis, or acute lung injury [18]. The immunomodulatory properties of MSCs were also shown to prevent transplant rejection in numerous studies; for example, MSC injection before skin transplantation in baboons prolonged graft survival [19]. 

MSCs were shown to be immunoprivileged. Since they are characterized by low levels of MHC-I and lack of MHC-II, they are not rejected by the host’s immune system [20]. Therefore, they could potentially replace traditional immunosuppressive agents in the case where their use is not recommended, limiting the activity of immune cells without eliciting the immune response themselves. However, another line of evidence shows that allogeneic MSCs do stimulate the immune response and can be rejected by recipient T cells [21]. It was also documented that MSCs can both inhibit and stimulate cytotoxic NK cells [22].

Aware of the controversy over the immunoprivileged nature of MSCs, in the present study we verified the impact of syngeneic or allogeneic BMSCs on skeletal muscle regeneration in an immunocompetent host (with special emphasis on inflammation). Furthermore, we also tested the effect of BMSC-conditioned medium injection into immunocompetent host regenerating muscles to determine which treatment would have the best outcome. We determined the impact of both types of cells and medium on various aspects of muscle regeneration, that is whole tissue structure, formation of new myofibers, formation of fibrosis, and immune cell infiltration. 

## 2. Materials and Methods

### 2.1. Animals

Animal care and all experimental procedures were approved by the Local Ethics Committee No.1 in Warsaw, Poland, according to the European Union Directive on the approximation in laws, regulations, and administrative provisions of the Member States regarding the protection of animals used for experimental and scientific purposes (permit number: 346/2017, LEC1, Poland). In cell transplantation experiments, 3-month-old males of the C57BL6N strain were used.

### 2.2. Cell Culture

The bone marrow cell population was obtained from the *tibialis* and *femoris* bones of 3-month-old C57BL6N males (syngeneic transplantation, referred to as SYN BMSCs) or Swiss-Tg (UBC-GFP) 30 Scha/J mice (allogeneic transplantation, referred to as ALLO BMSCs), as previously described [23]. The mice were obtained from the Animal Facility of the Faculty of Biology, University of Warsaw. Briefly, cells were washed out from bone marrow cavity using phosphate buffered saline (PBS) and centrifuged to remove erythrocytes. The cells obtained were seeded in gelatin coated plates in a medium composed of low glucose (1 g/L) Dulbecco’s modified Eagle’s medium (DMEM, Thermo Fisher Scientific, Waltham, MA, USA), 15% heat-inactivated fetal bovine serum (FBS, Thermo Fisher Scientific), 5000 units/mL of penicillin and streptomycin (Thermo Fisher Scientific), and maintained at 37 °C in a humidified atmosphere and 5% CO_2_. Cells were allowed to adhere for 48 h, after which the non-adherent ones were washed out with PBS. After obtaining a sufficient number, that is, 1 × 10^6^ cells (after 1 or 2 passages), cells were characterized by the presence of characteristic surface markers for MSCs, that is, CD105, CD73, and CD90. Briefly, cells were fixed with 3% PFA at room temperature for 10 min and then permeabilized with 0.1% Triton-X 100 (Sigma Aldrich, St. Louis, MO, USA) at room temperature for 5 min. Nonspecific antibody binding was blocked by incubation in 3% bovine serum albumin (BSA, Sigma-Aldrich) in PBS at room temperature for 1 h. Cells were then incubated at 4 °C overnight with primary antibodies: anti-CD105 (Abcam ab11414, diluted 1:1000, Cambridge, UK), anti-CD73 (Abcam ab175396, diluted 1:100), and anti-CD90 (Abcam ab225, diluted 1:100). Subsequently, cells were incubated with appropriate secondary antibodies conjugated with Alexa Fluor 594 or 488 (Thermo Fisher Scientific) and DRAQ5 (Biostatus Limited, Loughborough, UK) diluted 1:1000 at room temperature for 10 min. Cells were analyzed using the Axiovert 100M scanning confocal microscope with LSM 510 software ((Zeiss, Oberkochen, Germany). 

After confirmation that the cells obtained synthesized CD105, CD73, and CD90 and could therefore be classified as BMSCs, they were transplanted into the *gastrocnemius* muscle 24 h after cardiotoxin-induced injury. Prior transplantation, that is 48 h before injection into muscle, syngeneic BMSCs were labeled with VybrantTM DiI Cell Labeling Solution (Thermo Fisher Scientific).

### 2.3. Conditioned-Medium Collection 

After BMSCs reached confluence, the culture medium was switched to serum-free. Cells were cultured in 1 g/L of DMEM supplemented with 10% serum replacement (Thermo Fisher Scientific), 5 ng/mL of FGF-2 (R&D System), 2 mM of L-glutamine (Thermo Fisher Scientific), 0.1 mM ß-mercaptoethanol (Sigma Aldrich), and 5000 units/mL of penicillin and streptomycin (Thermo Fisher Scientific) for 48 h. The medium was then collected and centrifuged at 2000 rpm for 10 min to remove cellular debris. The collected supernatant was then sterilized with a 100 μm filter and used for transplantation or subjected to the Luminex assay (Austin, TX, USA). 

### 2.4. Skeletal Muscle Injury

After anesthesia of mouse (3-month-old male C57BL/6N) with isoflurane (AErrane, Baxter, Deerfield, IL, USA), *gastrocnemius* muscles were damaged by injection of 100 μM cardiotoxin (Latoxan, Biotechnology company, Portes-lès-Valence, France). After 24 h, the injured muscles were injected with 10 µL of 0.9% NaCl solution containing 1 × 10^6^ of either syngeneic or allogeneic BMSCs or 10 µL of BMSC-conditioned medium. In control experiments, the injured muscles were injected with 10 µL of 0.9% NaCl solution. After the procedure, the animals were kept in 14 h light:10 h dark regime with free access to food and water. Regenerating muscles were collected after 7, 14, or 30 days after injury, weighted, and frozen in isopentane chilled in liquid nitrogen and then stored for further analyses at −80 °C. Each variant of the experiment was carried out in at least three biological repetitions. 

### 2.5. Histological Analyses

The frozen mouse muscles were cut into 10 μm sections using cryostat (Microm HM505N, MICROM International GmbH, Walldorf, Germany). The cross sections were placed on slides and, after drying at room temperature, stored at 4 °C for further analyzes. For histological analysis, the sections were hydrated in PBS (10 min) and then stained with hematoxylin and eosin (Sigma Aldrich) or Masson’s Trichrome (Sigma Aldrich), according to the manufacturer’s instructions. The stained sections of each muscle were analyzed using a Nikon TE200 microscope and NIS Elements software (Nikon, Minato City, Tokyo). The area occupied by connective tissue in relation to the area of the entire section was determined using ImageJ 1.8.0 software (National Institutes of Health, Bethesda, MD, USA). For each experiment at least 20 sections were analyzed.

### 2.6. Immunohistochemical Analysis of Muscle Sections

Muscle sections were hydrated in distilled water, washed with PBS, and fixed in 4% paraformaldehyde in PBS at room temperature for 10 min. The samples were then washed twice with PBS, permeabilized with 0.3% Triton X-100 (Sigma Aldrich) in PBS at room temperature for 10 min and incubated in PBS containing 3% BSA (Sigma Aldrich), 2% donkey serum (DS; Sigma Aldrich) and 5% FBS at room temperature for 1 h. Next, all samples were incubated with primary antibodies: anti-CD45 (Abcam ab25386) or anti-CD68 (Abcam, ab53444) diluted in blocking buffer in PBS (1:100) at 4 °C overnight, followed by incubation with appropriate secondary antibodies conjugated with Alexa Fluor 488 or 594 (Thermo Fisher Scientific), diluted 1:200 in PBS with 3% BSA, 5% FBS, and 2% DS at room temperature for 1 h. The nuclei were visualized with Hoechst (Sigma-Aldrich) diluted 1:1000 in PBS for 10 min at room temperature. The samples were mounted with fluorescent mounting medium (Dako Cytomation, Glostrup, Denmark). The number of CD45+ or CD68+ cells was counted 14 days after cell transplantation. A total of 20 fields of view of 3 muscles from each experimental variant were analyzed with ImageJ software.

### 2.7. Optical Tissue Clearing and Whole Muscle Imaging in the Light-Sheet Microscope 

A separate batch of muscles was collected for optical imaging of the entire organ using the modified iDISCO+ method [24]. The detailed protocol is available at https://idisco.info (accessed on 1 December 2016). Briefly, the samples were first dehydrated at increasing concentrations of methanol (20–100%; at room temperature for 1 h each) and then stored at 4 °C in 1:3 methanol in dichloromethane (DCM). The samples were washed twice with 100% methanol and bleached with 1:5 H_2_O_2_ in a methanol solution at 4 °C. Next, they were rehydrated at decreasing concentrations of methanol (80–20%; 1 h each at room temperature) and permeabilized in 2% Triton X-100 for 1 h twice at room temperature. The samples were stored in a permeabilization solution (PBS/2% Tween-20/1.25 Triton X-100/1.15 glycine) for 24 h at 37 °C and in a blocking solution (PBS/1.68% Triton X-100/3% BSA/10% DMSO) for 48 h at 37 °C. The samples were then stained with anti-CD73 antibody (Abcam ab175,396, 1:200) or anti-GFP antibody (MBL, 598, 1:500) diluted in PBS/0.2% Tween-20 with 10 μg/mL heparin (PtwH) for 5 days at 37 °C. Then, they were washed with PtwH solution 4 times at room temperature and finally stained with the appropriate secondary antibody conjugated to Alexa 647 (1:500) diluted in PtwH for 5 days at 37 °C. The samples were then dehydrated at increasing concentrations of methanol (20–100%; 1 h each). The methanol was further washed out with DCM, first using a 1:2 methanol solution in DCM followed by two washes in pure DCM. Finally, the samples were placed in dibenzyl ether (DBE, Sigma Aldrich) for 1 week at 4 °C and then analyzed with a homemade light sheet microscope [25] equipped with an immersion objective LaVisionBioTec 4x NA 0.5, WD 6 mm (LaVision BioTec GmbH, Bielefeld, Germany), and Hamamatsu Orca-Flash4.0 camera (Spectra Services, Ontario, NY, USA). The 638 nm laser was used for excitation of fluorophores and 488 nm for excitation of tissue autofluorescence. Data were collected with 5-μm Z step and resolution in XY 1.44 × 1.44 μm. As the specimen was larger than the field of view of the microscope, imaging was performed as a series of 3D tiles (sequence of photos 800 × 1500 μm) that were later stitched, fused, and exported as a single tiff stack using TeraStitcher v 1.10.4 [26]. Segmentation to detect GFP positive cells was performed semi-automatically using Imaris (Bitplane Inc., Belfast, UK). 

### 2.8. RNA Isolation and qPCR Analysis

Total mRNA was isolated from the collected muscles, using a MirVana PARIS RNA and Native Protein Purification Kit (Thermo Fisher Scientific). Reverse transcription was performed using 1 μg total RNA and the RevertAid First Strand cDNA Synthesis Kit (Thermo Fisher Scientific), according to the manufacturer’s instructions. qPCR was performed using specific TaqMan^®^ (Thermo Fisher Scientific) probes (Supplementary Table S1) and the TaqMan Gene Expression Master Mix (Thermo Fisher Scientific). Data were normalized to *Hprt* expression level. The RNA isolated form mouse embryo at 13.5 days of development was used as a reference sample. Data were collected and analyzed with LightCycler 96 SW1.1 software (Roche, Basel, Switzerland). For each analysis, at least three independent experiments were performed. Ddct analysis was performed according to Livak and Schmittgen [27]. 

### 2.9. Luminex Assay

For protein isolation, the muscles were homogenized on ice with MirVana PARIS RNA and Native Protein Purification Kit (Thermo Fisher Scientific), according to the manufacturer’s protocol. Protein concentration was measured with the Pierce BCA Protein Assay Kit (Thermo Fisher Scientific), according to the manufacturer’s protocol. Subsequently, the obtained lysates were analyzed with the customized Mouse Magnetic Luminex Assay 10 Plex (Biotechne, Minneapolis, MN, USA) using the MAGPIX instrument (Luminex). A custom-made assay was used to analyze: CXCL1, CCL2, TNF-α, IGF-I, IL-4, IL-6, IL-10, IL-13, IL-33, and IFN-γ.

### 2.10. Statistical Analysis

All experiments were performed at least three times. Data are presented as mean ± standard deviation (SD), and one-way or two-way ANOVA with Tukey post hoc test was used for statistical analysis. Data found to be statistically significant are marked with asterisks (* *p* < 0.05; ** *p* < 0.01; *** *p* < 0.001; **** *p* < 0.0001).

## 3. Results

### 3.1. Factors Active during Muscle Regeneration Are also Secreted by Mouse BMSCs

**Figure 1:**
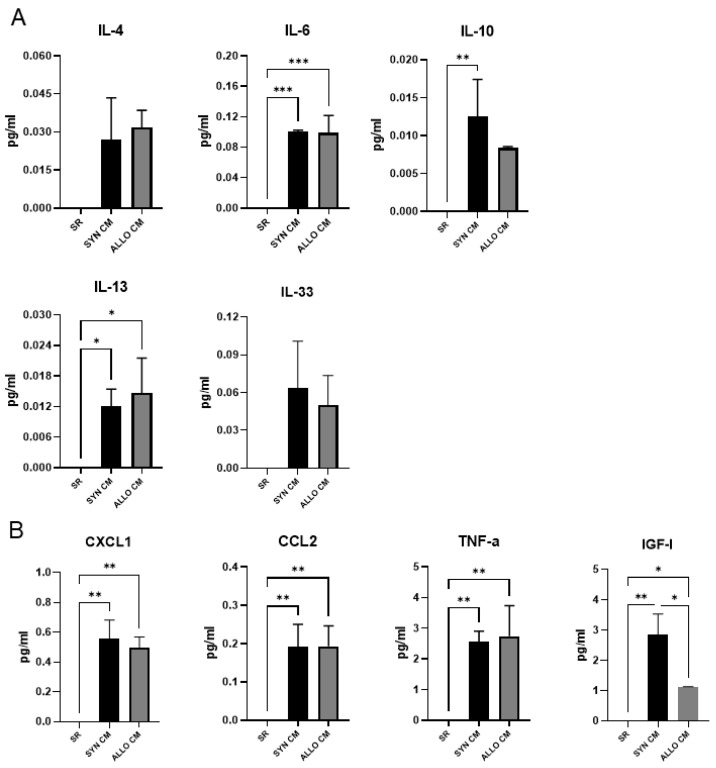
Luminex assay analysis of medium conditioned with SYN or ALLO BMSCs. Concentration of IL-4, IL-6, IL-10, IL-13, IL-33, CXCL1, CCL2, TNF-α, IGF-I in chemically defined medium and chemically defined medium conditioned for 48 h by BMSCs (CM). (**A**) Factors detected at low concentrations; (**B**) Factors detected at high concentrations. Data are presented as means of three independent experiments with standard deviations. Data found to be statistically significant are underlined on graphs and marked with asterisks (* *p* < 0.05; ** *p* < 0.01; *** *p* < 0.001).

In the initial set of experiments, by applying the Luminex assay, we determined the concentration of selected chemokines, cytokines, and growth factors in medium supplemented with SR and conditioned by BMSCs. The medium conditioned (referred to as CM) by syngeneic (SYN) or allogeneic (ALLO) BMSCs was collected after 48 h of the cell culture. Additionally, as a negative control, we used medium supplemented with SR but not conditioned by any cells. We analyzed CXCL1, CCL2, TNF-α, IGF-I, IL-4, IL-6, IL-10, IL-13, IL-33, and IFN-γ which are active and play a crucial role during muscle regeneration, as they are responsible for: attracting immune cells to the site of injury (CXCL1, CCL2), immune cell activation (TNF-α, IL-4, IL-6, IL-10, IL-13, IL-33 and IFN-γ) or new myofiber formation (IGF-I; [28]). We did not detect any of the analyzed proteins in the control medium. However, we detected high levels of most of them in CM, with the exception of IFN-γ that was not detected (data not shown) and IL-4, IL-10, IL-13, and IL-33, which levels were low, that is, below 0.10 pg/mL (Figure 1A). In comparison, IL-6 was detected at a concentration of 0.10 pg/mL (Figure 1A). The concentrations of the rest of the factors analyzed, that is the chemokines CXCL1 and CCL2, as well as TNF-α and IGF-I, were high (Figure 1B). 

We did not observe any significant differences in the levels of analyzed factors between medium conditioned by SYN or ALLO BMSCs (referred to as SYN CM or ALLO CM, respectively), apart from IGF-I, which level was higher in SYN CM than in ALLO CM. Thus, for transplantation experiments we used SYN CM.

### 3.2. SYN and ALLO BMSCs Survive in Regenerating Skeletal Muscle

**Figure 2:**
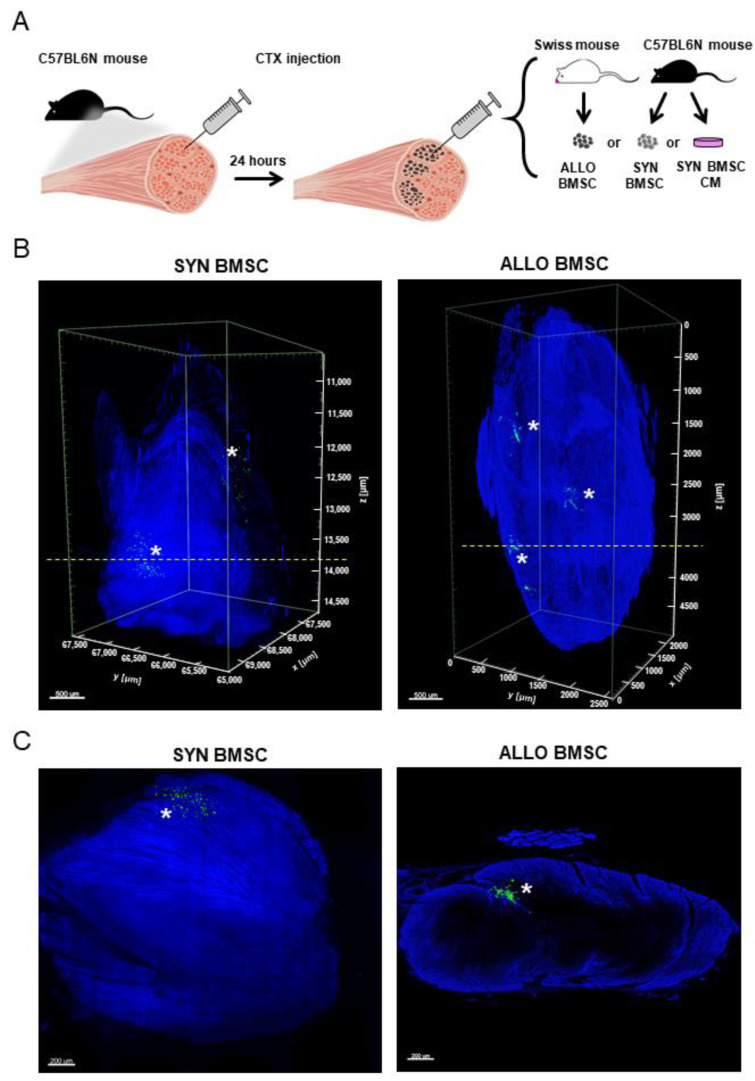
SYN or ALLO BMSC intramuscular injection. (**A**) Experimental procedure. (**B**) Whole muscle analysis and (**C**) optical sections of regenerating mouse skeletal muscles showing SYN and ALLO BMSC localization 14 days after muscle injury; images obtained with the iDISCO+ method; asterisks indicate localization of cells; SYN BMSCs or ALLO BMSCs are shown in green. Dashed lines visible on images in (**B**) indicate the plane of collection of optical sections shown in (**C**). For visualization of the entire muscle, myoglobin autofluorescence was collected (for each muscle treatment *n* = 2).

After confirming that isolated cells synthesized MSC surface markers (CD105, CD73, and CD90, data not shown) and could be classified as BMSCs we injected 1 × 10^6^ of either SYN or ALLO BMSCs into the *gastrocnemius* muscle 24 h after cardiotoxin-induced injury. In the first step, we determined whether both types of cells, that is SYN or ALLO BMSCs, were able to survive in the recipient muscles at least up to 14 days after injury (experimental scheme presented in Figure 2A). To be able to visualize the injected cells, we used BMSCs constitutively expressing GFP (ALLO) or DiI-labelled cells (SYN). Since our main objective was to localize cells within the whole muscle, we used the iDISCO+ technique that enabled 3D imagining with high image quality and resolution. Since DiI dimmed with time, to ensure precise localization of transplanted cells in the recipient’s tissue, we also stained the muscles with anti-CD73 antibody. In addition, we analyzed muscle autofluorescence to visualize the structure and boundaries of the tissue (Figure 2B). We confirmed the presence of both SYN and ALLO cells in the *gastrocnemius* muscle analyzed at 14 day after injury. However, the observed cells grouped in few regions of the muscle, what suggested poor migration (Figure 2B). We did not observe significant differences in cell dispersion between muscles injected with either SYN or ALLO BMSCs (Figure 2B).

### 3.3. SYN BMSC Conditioned Medium Limits, while the Presence of ALLO BMSCs Increases the Fibrosis Formation

**Figure 3:**
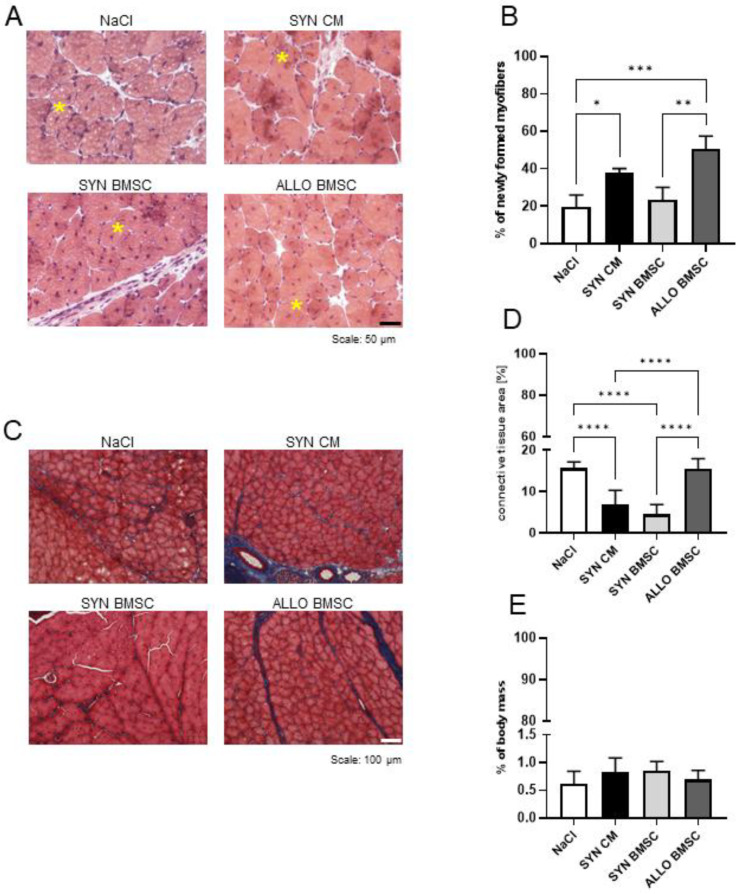
Regeneration of mouse skeletal muscles injected with NaCl or SYN CM or SYN or ALLO BMSCs. (**A**) Hematoxylin-eosin-stained sections of regenerating muscles injected with NaCl, SYN CM, SYN or ALLO BMSCs, analyzed on day 14 after injury. Representative newly formed myofibers with centrally localized nuclei are marked with yellow asterisks. Scale bar: 100 μm. (**B**) Numbers of newly formed myofibers in the regenerating muscle injected with NaCl or SYN CM or SYN or ALLO BMSCs, analyzed on day 14 after injury. (**C**) Masson’s trichrome stained sections of regenerating muscles injected with NaCl or SYN CM or SYN or ALLO BMSC, analyzed on day 14 after injury. Skeletal muscle in pink, connective tissue in blue. Scale bar: 100 μm. (**D**) Proportion of connective tissue area to whole muscle area in regenerating muscle injected with NaCl or SYN CM or SYN or ALLO BMSCs analyzed on day 14 after injury. (**E**) Weight of regenerating muscles injected with NaCl or SYN CM or SYN or ALLO BMSCs, analyzed on day 14 after injury, shown in proportion to mouse weight. Data found to be statistically significant are underlined on graphs and are marked with asterisks (* *p* < 0.05; ** *p* < 0.01; *** *p* < 0.001; **** *p* < 0.0001).

In the next step, we determined how the injection of SYN or ALLO BMSCs or SYN CM influenced the structure of the regenerating muscle. Muscles injected with NaCl served as a control. By analyzing hematoxylin-eosin-stained sections, we assessed the number of newly formed myofibers in regenerating muscles 14 days after cardiotoxin-induced injury. Myofibers with centrally located nuclei were visible in all analyzed muscles, confirming the progression of muscle regeneration (Figure 3A). Analysis of the number of newly formed myofiber revealed that it was significantly higher in the muscles injected with ALLO BMSCs or SYN CM than in the muscles injected with SYN BMSCs or NaCl (Figure 3B). 

To assess the level of fibrosis, that is, extensive connective tissue development that hinders muscle function, we analyzed histological sections stained with Masson’s trichrome (Figure 3C). The proportion of fibrosis area to the whole muscle area was significantly lower in muscles injected with SYN BMSCs or SYN CM than in other muscle groups (Figure 3D). Importantly, the level of fibrosis was more than twice as high in the muscle injected with NaCl- or ALLO-BMSCs [15.63% (+/−1.52%) and 15.43% (+/−2.45%), respectively] compared to those injected with SYN-BMSCs and SYN-CM [4.44% (+/−2.78%) and 6.90 (+/−3.38%), respectively]. Despite the differences described, the mass of the regenerating muscles, when calculated in relation to the total body mass, was similar in all groups (Figure 3E).

### 3.4. SYN CM Decreases Significantly, while the Presence of ALLO BMSCs Increases Inflammation in Regenerating Muscles

In the next step, we determined the number of immune cells that infiltrate regenerating muscle, as well as assessed the level of selected immune cell markers. We analyzed mRNAs encoding *Cd68* (M1 macrophage marker), *Cd163* (M2 macrophage marker), *Ccr7* (T lymphocyte marker), and *Ly6c2* (neutrophil marker) in muscles collected on day 14 after injury, when the inflammation stage should already be finished and the formation of new myofiber is proceeding. 

**Figure 4:**
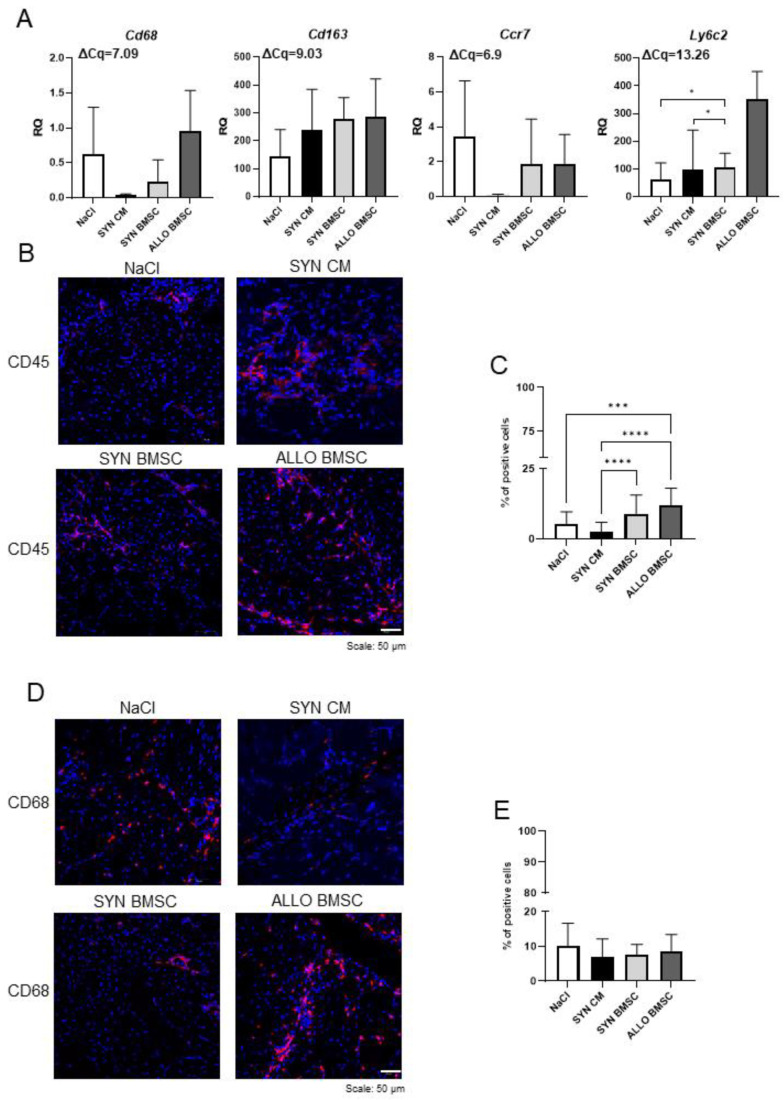
Analysis of immune cell infiltration in regenerating muscles injected with NaCl or SYN CM or SYN or ALLO BMSCs. (**A**) Expression of immune cell markers: *Cd68*, *Cd163*, *Ccr7,* and *Ly6c2*. *Hprt* was used as a reference gene. RQ = 1 for the level of gene expression detected in 13.5-day-old mouse embryo (used as reference sample). Average ΔCq for the reference sample is shown in each graph. Data are presented as means of three independent experiments with standard deviations. (**B**) Representative images of CD45 immunostaining in the muscles analyzed 14 days after injury. Analyzed proteins are shown in red; DNA in blue. Scale bar: 50 µm. (**C**) Proportion of CD45 positive cells to all cells counted in sections of analyzed muscles 14 days after injury. For all variants *n* ≥ 20. (**D**) Representative images of CD68 immunostaining in the muscles analyzed 14 days after injury. Analyzed proteins are shown in red; DNA is shown in blue. Scale bar: 50 µm. (**E**) Proportion of CD68-positive cells to all cells counted in analyzed muscle sections 14 days after injury. For all variants *n* ≥ 20. Data found to be statistically significant are underlined on graphs and are marked with asterisks (* *p* < 0.05; *** *p* < 0.001; **** *p* < 0.0001).

The *Cd68* and *Ccr7* transcripts were detected in all muscle groups analyzed, however, their levels were very low in SYN CM-injected muscles (Figure 4A). *Cd163* mRNA was detected at a relatively high level in all muscle groups without significant differences between them (Figure 4A). *Ly6c2* was also detected in all muscles studied; however, its level was low with the exception of ALLO BMSC injected muscles characterized by relatively high expression of this marker (Figure 4A). 

Next, we determined the number of CD45 and CD68 positive cells in all muscle groups on day 14 after injury. CD45 positive cells were present in all types of muscles analyzed (Figure 4B). The number of CD45 positive cells was significantly higher in ALLO BMSC-injected muscles than in all other counterparts (Figure 4C). At the same time, we observed that this cell population was scarce in the muscles injected with SYN CM (Figure 4C). We also determined the number of CD68 positive M1 macrophages, which play a crucial role in muscle regeneration, mediating the clearance of fibrosis [29,30]. CD68 positive cells were found in all muscle groups, but no significant differences were observed between them (Figure 4D, E).

### 3.5. The Presence of ALLO BMSCs Increases the Level of Pro-Inflammatory Factors in Regenerating Muscles

Subsequently, we looked at the concentration of selected chemokines, cytokines, and growth factors in all groups of regenerating muscles. Using the Luminex technique, we determined levels of pro-inflammatory factors, such as: CXCL1, CCL2, TNF-α, IL-4, IL-6, IL-13, and IL-33, as well as the anti-inflammatory, such as: IL-10 and IGF-I [28] in muscles collected at three time points: (1) on day 7 after cardiotoxin-induced injury (when damaged tissue is removed but new myofibers have not yet formed), (2) on day 14 (when new myofibers are already present), and (3) on day 30 (when muscle structure is already restored). 

**Figure 5:**
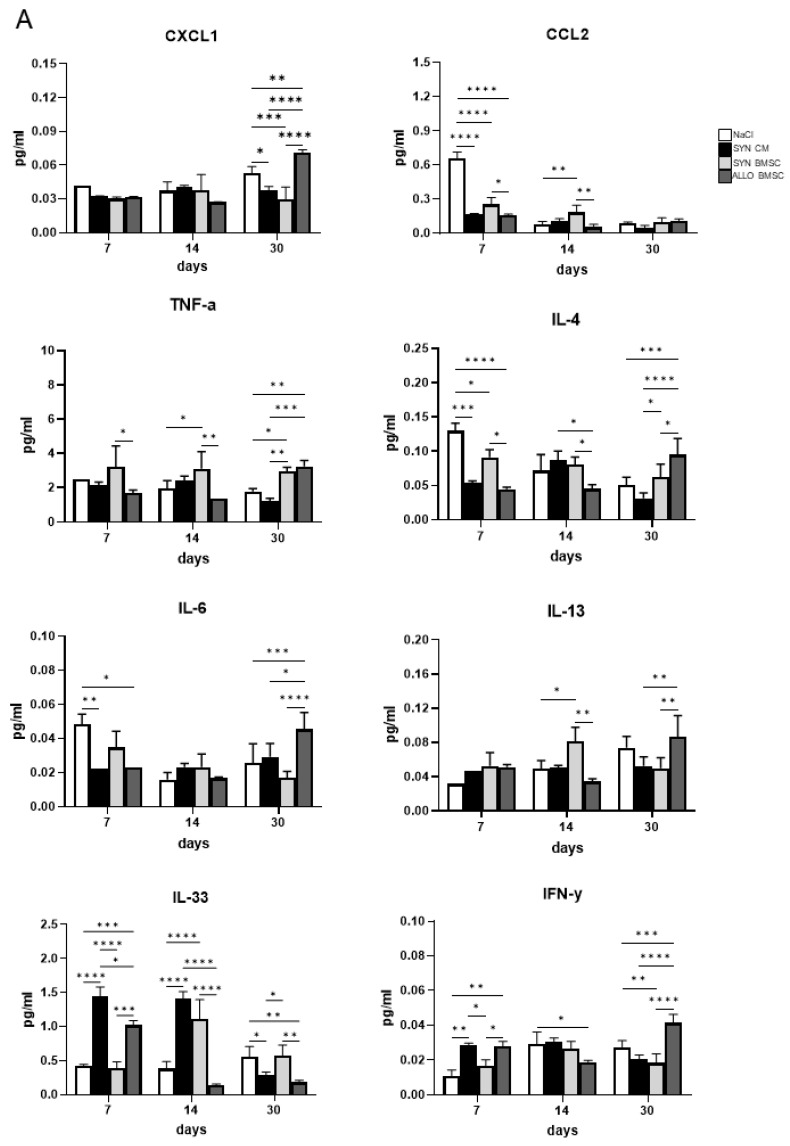

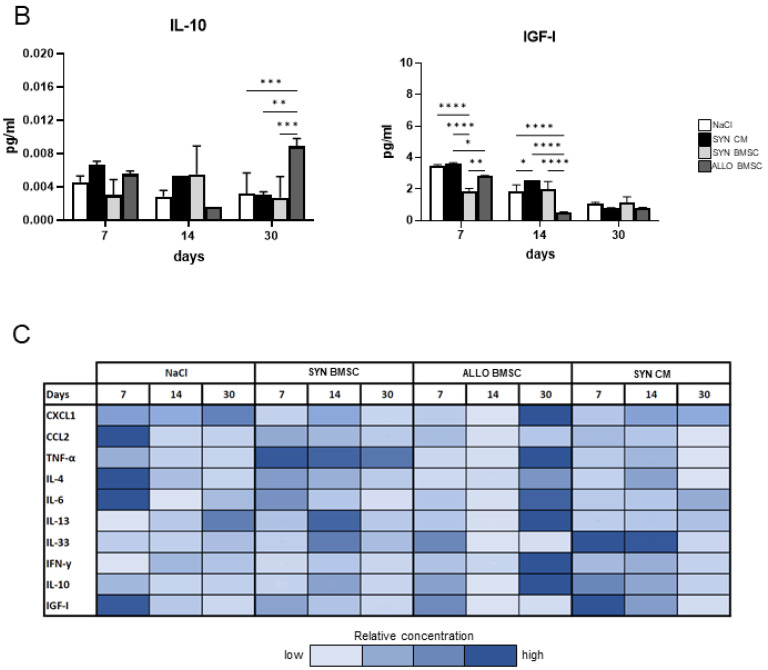
Luminex assay analysis of regenerating muscles injected with NaCl or SYN CM or SYN or ALLO BMSCs. (**A**) Concentration of pro-inflammatory factors: CXCL1, CCL2, TNF-α, IL-4, IL-6, IL-13, IL-33, and IFN-γ in muscles injected with NaCl (control, white bars) or SYN CM (black bars) or SYN BMSCs (light grey bars) or ALLO BMSCs (dark grey bars) on days 7, 14, and 30 after injury. (**B**) Concentration of anti-inflammatory factors: IL-10 and IGF-I in muscles injected with NaCl (control, white bars) or SYN CM (black bars) or SYN BMSCs (light grey bars) or ALLO BMSCs (dark grey bars) on days 7, 14, and 30 after injury. Data are presented as means of three independent experiments with standard deviations. (**C**) Graphical summary of the Luminex assay analysis. The darker hue represents a higher relative concentration for each factor compared to the sample in which the highest concentration was found. Data found to be statistically significant are underlined on graphs and are marked with asterisks (* *p* < 0.05; ** *p* < 0.01; *** *p* < 0.001; **** *p* < 0.0001).

Analyzing CXCL1 levels, we did not observe differences between treatments on day 7 and 14, while on day 30 after injury the concentration of this chemokine in muscles injected with NaCl (control variant) or ALLO BMSC was significantly higher than in muscles injected with SYN BMSCs or SYN CM (Figure 5A). A different pattern was observed for CCL2, the level of which was the highest on day 7 in NaCl-injected muscles, compared to other muscle groups. On day 14, the level of CCL2 was higher in the muscles injected with SYN BMSCs than in the muscles injected with NaCl or ALLO BMSCs, but on day 30 its level was negligible in all muscle groups (Figure 5A). The level of this factor decreased significantly during regeneration in all muscles analyzed (Supplementary Figure S1). 

TNF-α level was significantly higher in the SYN BMSC injected muscles than in the ALLO BMSC injected ones on days 7 and 14. However, on day 30 the level of this cytokine was similar in these muscle groups and significantly higher, compared to those injected with NaCl or SYN CM (Figure 5A). Importantly, the level of TNF-α was elevated at this stage of regeneration only in ALLO BMSC injected muscles (Supplementary Figure S1). On day 7 the level of IL-4 was significantly lower in all muscle groups, compared to the control (Figure 5A). Importantly, on day 30 the level of IL-4 was significantly higher in ALLO BMSC-injected muscles compared to other muscle groups (Figure 5A). ALLO BMSC-injected muscles were the only ones characterized by an increase in the level of IL-4 during regeneration since the expression of this factor decreased significantly in all other experimental variants (Supplementary Figure S1). 

On day 7, the level of IL-6 was significantly lower in ALLO BMSC and SYN CM injected muscles than in control, but no significant differences between all variants were observed on day 14. Interestingly, on day 30 the level of IL-6 was significantly higher in ALLO BMSC-injected muscles compared to other muscle groups (Figure 5A) and again increased only in this muscle group during regeneration progression (Supplementary Figure S1). We did not observe any significant differences in the level of IL-13 between all treated muscles on day 7, while on day 14 it was significantly higher in the muscles injected with SYN BMSCs than in the control and the ALLO BMSC injected. On day 30 we found a significantly higher level of IL-13 in ALLO-injected muscles than in other treated muscles, that is, muscles injected with SYN BMSCs and SYN CM (Figure 5A and Figure S1). 

IL-33 was significantly increased in SYN CM-injected muscles than in other muscle groups on day 7 after injury. At this time point, the level of IL-33 was also elevated in SYN BMSC-injected muscles, while on day 14 in both SYN BMSC- and SYN CM-injected muscles, compared to those injected with NaCl or ALLO BMSC. On day 30 a significantly lower level of IL-33 was found in ALLO BMSC and SYN CM injected muscles than in other muscle groups (Figure 5A). However, the level of IL-33 decreased in all analyzed muscles as regeneration progressed (Supplementary Figure S1). The level of IFN-γ was substantially higher in ALLO BMSC and SYN CM injected muscles compared to other muscle groups on day 7. On day 14, its level was lower in ALLO BMSC injected muscles compared to NaCl-treated control, but on day 30 IFN-γ was, in contrast, significantly higher in ALLO BMSC injected muscles compared to all other variants (Figure 5A). Importantly, only in ALLO BMSC treated muscles IFN-γ level increased significantly at that late stage of regeneration (Supplementary Figure S1). 

Next, we determined the level of anti-inflammatory factors, i.e., IL-10 and IGF-I. No significant differences in IL-10 level were detected on days 7 and 14. However, on day 30 IL-10 concentration was higher in ALLO BMSC injected muscles than in all other experimental groups (Figure 5B). The level of IGF-I was substantially lower in SYN BMSC injected muscles on day 7 compared to day 14 in the ALLO BMSC injected. No differences were observed on day 30 (Figure 5B). 

In summary, we showed that the level of most of the pro-inflammatory factors analyzed (such as CXCL1, TNF-α, IL-4, IL-6, and IFN-γ) was significantly higher in ALLO BMSC injected muscles after 30 days of regeneration, while the level of IGF-I, which is significant for the formation of new myofibers, increased markedly in muscles treated with SYN CM on days 7 and 14, when this process occurs (summary of the analysis of the Luminex assay in Figure 5C).

## 4. Discussion

Throughout the years, it has been clear that BMSCs may serve as a source of mesodermal derivatives, such as chondrocytes or osteoblasts [31,32]. Furthermore, it was shown that they can fuse with myoblasts and incorporate into newly formed myofibers, although to a very limited extent [33]. In mice, it was also shown that the myogenic potential of BMSCs, defined as the ability to convert into myogenic cells, can be promoted by overexpression of genes encoding NICD (Notch Intracellular Domain), β-CATENIN, PAX3 or miRNAs involved in muscle regeneration [34,35]. Despite the limited naïve myogenic potential, BMSCs are still considered in terms of therapy for dysfunctional muscles due to their other properties, that is as immunomodulatory agents. 

In previous studies, BMSCs were shown to decrease NK cell activation and cytotoxicity in vitro, as well as T lymphocyte proliferation by direct cell-cell contact or by secreted factors, such as prostaglandins [17]. In clinical trials, autologous BMSCs are the first choice for transplantation in humans as they are characterized by a lower risk of immune rejection. However, obtaining a sufficient number of cells to be transplanted is not always possible. On the other hand, allogeneic BMSCs are usually easily available as they can be isolated from several donors, but the risk of immune rejection is higher. These two strategies, that is the application of autologous or allogeneic BMSCs were compared during many already conducted clinical trials [36,37]. In many cases, for example, in treatments for cardiomyopathies or bone and cartilage repair, no substantial differences were observed [38]. Under some conditions, a better therapeutic outcome was observed after allogeneic BMSC treatment, as, for example, in the case of patients suffering from lupus nephritis [39]. Autologous BMSCs collected from them were characterized by abnormal levels of cytokines and their infusion led to limited clinical improvement, while allogeneic BMSC infusion resulted in a decrease in serological autoimmune markers [39,40]. However, another line of evidence revealed that despite their immunomodulatory properties, allogeneic MSCs can trigger immune rejection, especially after repeated transplantations [21,41,42]. Since BMSCs are considered immunoprivileged in the current study we asked whether autologous (syngeneic) or allogeneic BMSC transplantation into an immunocompetent host would have a similar or significantly different impact on muscle regeneration with special emphasis on inflammation. Moreover, knowing that the action of BMSC is mediated primarily by paracrine factors, we assessed the impact of conditioned medium injection on muscle regeneration as well as the level of selected factors important for the progression of muscle regeneration. This analysis revealed that syngeneic and allogeneic BMSCs cultured under standard culture conditions synthesized many factors essential for muscle regeneration, that is CXCL1, CCL2, TNF-α, IGF-1, IL-6, IL-10, and IL-13. This finding is consistent with other reports documenting that factors, such as IL-6, CXCL1, and IGF-I, were present in the secretome of MSCs isolated from bone marrow or adipose tissue, although the level of these proteins varied between donors [43,44].

BMSCs, both autologous and allogeneic, were shown to have limited engraftment ability after systemic delivery [45]. For this reason, we injected cells, syngeneic, or allogeneic, directly into skeletal muscle. Since cells transplanted to injured muscles are often characterized by a poor survival rate due to a high apoptosis rate [46,47], we first confirmed that the BMSCs used in our experiments were able to survive in regenerating muscles and were indeed present in the tissue as reconstruction proceeded. However, as we showed, their ability to migrate was poor, as it was visualized using iDISCO+ method. This tool was already used to visualize intramuscular innervation in skeletal muscle [48] and skeletal muscle tissue in teratomas [8]. In the current study, we show that it can also be applied successfully to analyze the localization of injected cells into muscles, including their potential incorporation into newly formed myofibers. Importantly, this 3D imaging technique allows tracking scarce cell populations that could be overlooked when analyzing muscles using different methods, such as cryosectioning.

Recent observations indicate that syngeneic or allogeneic BMSCs and SYN CM can improve muscle structure after acute and chronic muscle injury [49]. In our study we observed a higher number of newly formed myofibers in muscles injected with ALLO BMSCs or SYN CM, yet muscles injected with ALLO BMSCs were also characterized by the larger area of fibrosis, higher levels of expression of immune cell markers, and higher number of CD45 positive cells. Andrade and colleagues also showed that intramuscular injection of allogeneic BMSC increased the formation of new myofibers, however, the authors examined neither fibrosis nor inflammation in treated muscles [50] Although the presence of immune cells is critical for the clearance of damaged tissue during the degeneration phase and SC activation in regenerating muscle, their persistence in the tissue during the reconstruction phase promotes fibrosis and hinders muscle reconstruction [51,52].

We found that the level of several proinflammatory factors that are important for proper muscle regeneration progression was significantly elevated in ALLO BMSC injected muscles, while decreased in the SYN CM injected. In the case of ALLO BMSC-injected muscles, it was especially visible in the later stages of muscle regeneration, again indicating prolonged inflammation and impaired tissue regeneration. On the other hand, muscles injected with SYN CM were characterized by higher concentrations of IGF-I, which for example promotes the formation of new fibers [53]. Furthermore, IGF-I was shown to promote macrophage polarization into M2 phenotype [54], therefore, its high level in this experimental setting can also contribute to the limited fibrosis and enhanced myofiber formation observed in muscles injected with SYN CM. Consistent with our observations, the study by Luo and co-workers also confirms that BMSC-derived exosomes limit muscle fibrosis by promoting macrophage polarization into anti-inflammatory M2 macrophages [55]. Our experiments also revealed higher levels of IL-33 in muscles injected with SYN CM. This cytokine is most commonly classified as a pro-inflammatory cytokine, but it was reported that in skeletal muscle it can also promote muscle growth [56]. 

In the previously published study, we showed that syngeneic murine adipose-derived stromal cells (ADSCs) decreased the level of Il-6 mRNA in muscle, compared to NaCl injected muscles [57]. We did not observe such an effect in muscle injected with SYN BMSCs, which could be possibly explained by the differences between ADSCs and BMSCs, extensively commented on elsewhere [58,59]. The level of selected pro- and anti-inflammatory cytokines in muscles injected with BMSCs was also studied by Helal and coworkers [60]. Using a rat model, they showed that injection of SYN BMSCs into the injured muscles decreased the concentration of proinflammatory factors, such as IL-1, IL-6, and TNF-α, while increasing the concentration of IL-10 [60]. In our study, we did not observe such an effect in the muscles injected with SYN BMSCs. The observed discrepancies could be attributed, apart from inter-species differences, to the different phases of the regeneration examined. Helal and colleagues looked at the cytokine concentration only at the later stages (28 days after muscle injury), while we looked at muscles collected at three time points when different phases of regeneration occur. However, both mentioned studies also confirm our observation that the presence of MSCs in skeletal muscles significantly modulates levels of pro- and anti-inflammatory cytokines in the regenerating muscle. However, our study also clearly shows that injection of CM is sufficient to achieve the same effect, meaning that the presence of cells is not crucial for it.

In summary, in the present study we verified which treatment, injection with ALLO BMSCs or SYN BMSCs or BMSC CM, would be the best approach, manifested by reduced inflammation, limited fibrosis, and proper muscle reconstruction. Combined analysis of muscle histology, immune cell infiltration, and levels of selected chemokines, cytokines, and growth factors important for muscle regeneration showed that injection with BMSC CM is the most beneficial strategy, as it resulted in increased formation of new fibers, which may be connected, that is with elevated levels of IGF-1. In addition, in SYN CM-injected muscles, both the development of inflammation and fibrosis were reduced. Compared to other studies, the injection of SYN BMSCs, although not detrimental to muscle regeneration, did not result in such a significant improvement in this process. Therefore, considering the limitations in obtaining BMSCs and their propagation, this approach does not appear to be optimal in terms of dysfunctional skeletal muscle therapies. Finally, transplantation of ALLO BMSCs to injured muscles resulted in a visibly increased immune response, which hindered regeneration by promoting connective tissue formation. Therefore, although ALLO BMSCs can survive in regenerating muscles of an immunocompetent host, their transplantation is characterized by a significantly higher risk of extensive inflammation and impairment of muscle reconstruction. Therefore, this approach does not appear to be the most promising in terms of immunosuppression, while developing optimal cell therapy for dysfunctional skeletal muscles.

## Data Availability

Not applicable.

## Supplementary Materials

The following supporting information can be downloaded at: https://www.mdpi.com/article/10.3390/cells11182843/s1, Figure S1: Concentration of selected pro-inflammatory and anti-inflammatory factors in muscles injected with either NaCl, SYN CM, SYN BMSCs or ALLO BMSCs. Table S1: Probes used in qPCR analysis.
