# Supplementary material for: Comparison of Muscle Regeneration after BMSC-Conditioned Medium, Syngeneic, or Allogeneic BMSC Injection
Source: Cells. 2022 Sep 12;11(18):2843. doi: 10.3390/cells11182843 (PMC9497150; doi:10.3390/cells11182843)
Supplement: Supplementary file 1 [file cells-11-02843-s001.zip › cells-1806188-supplementary.pdf]

Supplementary Materials:

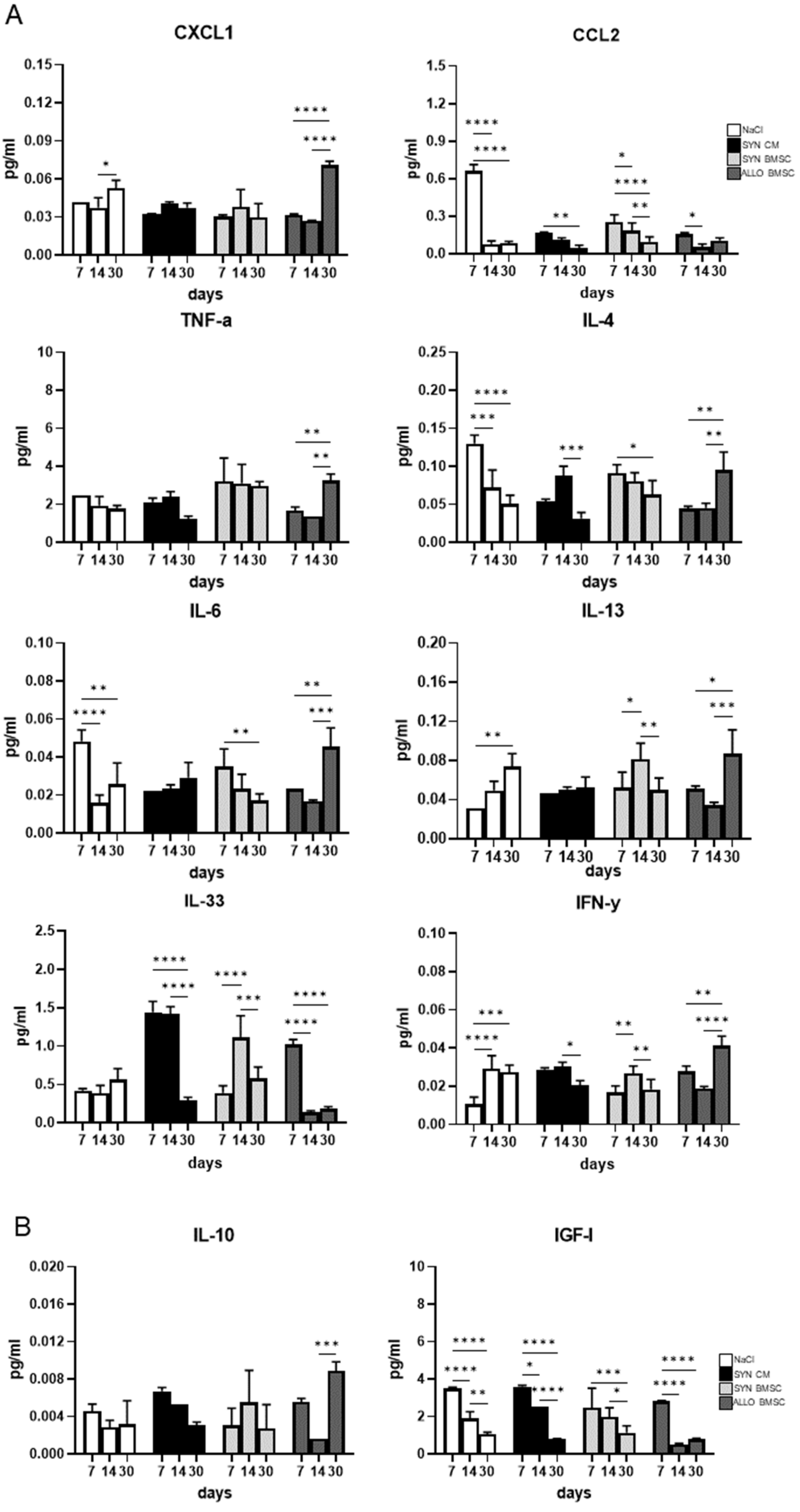

Supplementary Figure S1. Concentration of selected pro-inflammatory factors in muscles injected with either NaCl, SYN CM, SYN BMSCs or ALLO BMSCs. (A) Concentration of pro-inflammatory factors: CXCL1, CCL2, TNF-α, IL-4, IL-6, IL-13, IL-33, and IFN-γ in muscles injected with NaCl (control, white bars) or SYN CM (black bars) or SYN BMSCs (light grey bars) or ALLO BMSCs (dark grey bars) on day 7, 14 and 30 after injury. (B) Concentration of anti-inflammatory factors: IL-10 and IGF-I in muscles injected with NaCl (control, white bars) or SYN CM (black bars) or SYN BMSCs (light grey bars) or ALLO BMSCs (dark grey bars) on day 7, 14 and 30 after injury. Data are presented as means of three independent experiments with standard deviations. Data found to be statistically significant are underlined on graphs and are marked with asterisks (* $p < 0.05$; ** $p < 0.01$; *** $p < 0.001$; **** $p < 0.0001$).

Supplementary Table S1. Probes used in qPCR analysis.

Gene name	Probe number	Marker
Ccr7	Mm01301785	T lymphocytes
Cd68	Mm03047340	M1 macrophages
Cd163	Mm00474091	M1 macrophages
Ly6c2	Mm00841873	M1 macrophages
Hprt	Mm00446968_m1	M1 macrophages
